# Tourism image classification based on convolutional neural network SqueezeNet——Taking Slender West Lake as an example

**DOI:** 10.1371/journal.pone.0295439

**Published:** 2024-01-29

**Authors:** Lantao Xu, Xuegang Chen, Xinlu Yang

**Affiliations:** 1 School of Geographical Science and Tourism, Xinjiang Normal University, Urumqi, Xinjiang, China; 2 College of Foreign languages, Xinjiang Normal University, Urumqi, Xinjiang, China; GIET University, INDIA

## Abstract

Tourism image classification plays an important role in the study of clarifying the real perception of tourism resources by tourists, which cannot be studied in depth by human vision alone. The development of convolutional neural networks in computer vision brings new opportunities for tourism image classification research. In this study, SqueezeNet, a lightweight convolutional neural network, was selected and improved on the basis of the original model for 3740 Slender West Lake tourism image datasets. It is found that the validation accuracy of the model is up to 85.75%, and the size is only 2.64 MB, which is a good classification effect. This reduces the parameters while ensuring high accuracy classification of tourism images, providing a more scientific reference for the study of tourism images and pointing out a new direction for the development and planning of tourism resources.

## 1 Introduction

Image is an important carrier of human perception of the world and a discourse metaphor of visual image [1]. From its spatial, chromatic, and formal representations, human beings can derive a multitude of visual experiences. Images have consistently played a crucial role in various social and cultural contexts. The pursuit of visual impact has been an ongoing endeavor since the early days of primitive human rock wall paintings. Prior to the advent of photography, ancient images primarily served as aesthetic objects in the form of paintings. Renaissance paintings, for instance, captured fleeting scenes, including portraits and landscapes. The integration of art and science is evident in Leonardo Da Vinci’s Mona Lisa, which showcases humanistic thoughts and realistic techniques. Chinese painting art has a deep and rich history, spanning thousands of years and documenting various aspects of Chinese culture. For instance, pre-Qin period frescoes, bronze decorations, and silk paintings record myths, legends, and historical figures. In the Song Dynasty, Zhang Zeduan’s masterpiece ’Along the River at Qingming Festival’ vividly portrays the prosperous society of that time. The invention of the first daguerreotype camera in the 1830s by Frenchman Daguerre was based on the photographic optical principle of ’keyhole imaging’ discovered by Mozi during the Spring and Autumn Period and the Warring States Period [2]. Since then, from color photos to digital photos, and then to the perfect combination of mobile phones and cameras in the early 21st century, the maturity of photography technology has made people’s visual experience more and more rich, and people have higher and higher requirements for images. Modern people also extensively use photography technology in tourism activities, with the help of tourism images to record the moment of truth, goodness and beauty in tourism activities, in order to pursue visual stimulation and deepen the attachment to tourism places. Travel photography has become more convenient in the Internet era, and tourists are used to uploading photos taken during travel to online platforms [3]. These photos document the tourists’ activities such as eating, living, traveling, shopping, and entertainment. They provide a direct representation of the tourists’ behavior, intentions, and other information while observing the natural or cultural landscape [4]. Compared with online comments, tourism images are more objective and persuasive. However, since tourism images are not easy to be quantified, they are mostly used as resources for tourism interpretation and auxiliary tools for tourist comments. Moreover, it is difficult for researchers to fully identify details in images by relying on human vision alone, and there are defects such as focusing only on significant content, subjective description and limited processing of the same task [5]. Therefore, it is necessary to find a more scientific technology to replace human vision to realize the study of tourism images.

With the vigorous development of the field of artificial intelligence, computer vision has been widely used in satellite remote sensing [6], engineering inspection [7], medical imaging [8], meteorological monitoring [9], unmanned driving [10], face recognition [11] and other aspects. The research of computer vision in digital image is mainly divided into two modules: semantic perception and geometric attribute. Semantic perception includes image classification [12], object detection [13], image segmentation [14], image retrieval [15], etc. Geometric properties include 3D modeling [16], binocular vision [17] and augmented reality [18]. In addition, in recent years, the application of deep learning in the field of computer vision is more and more hot. Deep learning is a method to acquire characteristic experience to learn complex concepts in the process of giving a large amount of data to a computer and making it hierarchical. The image classification technique based on Convolutional Neural Network (CNN) has triggered a research upsurge by scholars at home and abroad. In this regard, the research level of foreign scholars is relatively rich, mainly focusing on the classification of satellite image, architectural image, weather image and vehicle image. Emmanuel et al. proposed different iterative enhancement algorithms on the basis of CNN to improve the quality of satellite image classification [19]. Reza et al. classified architectural images of different geographical locations based on CNN, and the transfer learning model can also accurately identify urban roads, trees and other landscapes [20]. In meteorology research, Cewu et al. used deep learning, support vector machine (SVM) and other methods to build a mixed model to distinguish sunny and cloudy weather images [21]. In addition, since cars are usually involved in criminal acts such as trafficking, Nicolas et al. compared the robustness of different CNN architectures with other inspection and evaluation methods in order to replace the manual inspection link in transportation, and found that CNN method was superior, so as to realize the identification of hidden cars in X-ray images of goods based on classification, so as to arrest criminals [22]. For this kind of research, domestic scholars also have different degrees of attainments. Xue Zhaohui et al. extended deformable convolution from spatial dimension to spectral dimension, designed spectral deformable convolution, and experimentally proved the effectiveness of this method in realizing hyperspectral image classification [23]. Zhu Yonghong et al. proposed an improved CNN framework for the classification of flame images of ceramic shuttle kiln to improve the classification effect [24]. Zhang Cheng et al. used CNN to extract the color features of three-channel concrete image, and integrated grid search algorithm optimized support vector machine for image classification, thus increasing the classification accuracy [25]. Sun Dongyang et al. used AlexNet, MobileNetV3 and ResNet50 image classification convolutional neural networks for transfer learning to classify Marine pasture reef biological images, and the experiment proved that the three methods have good classification effect [26]. In the study of agricultural intelligent recognition of insect species, Wei Fuyu et al. carried out transfer learning based on various image classification techniques under CNN and compared the training results to provide reference for the application of insect species scene [27]. Regarding the problem of road congestion detection, Quandian Juan et al. used lightweight convolutional neural network to classify vehicle density images, providing technical reference for traffic monitoring systems [28]. To sum up, there are abundant researches on image classification based on convolutional neural network at home and abroad, but foreign scholars have a significant awareness of innovation in this framework, and domestic scholars have a large room for improvement in this kind of research. However, it is noteworthy that domestic and foreign scholars have little research on tourism image classification.

## 2 Research area and data source

**Fig 1:**
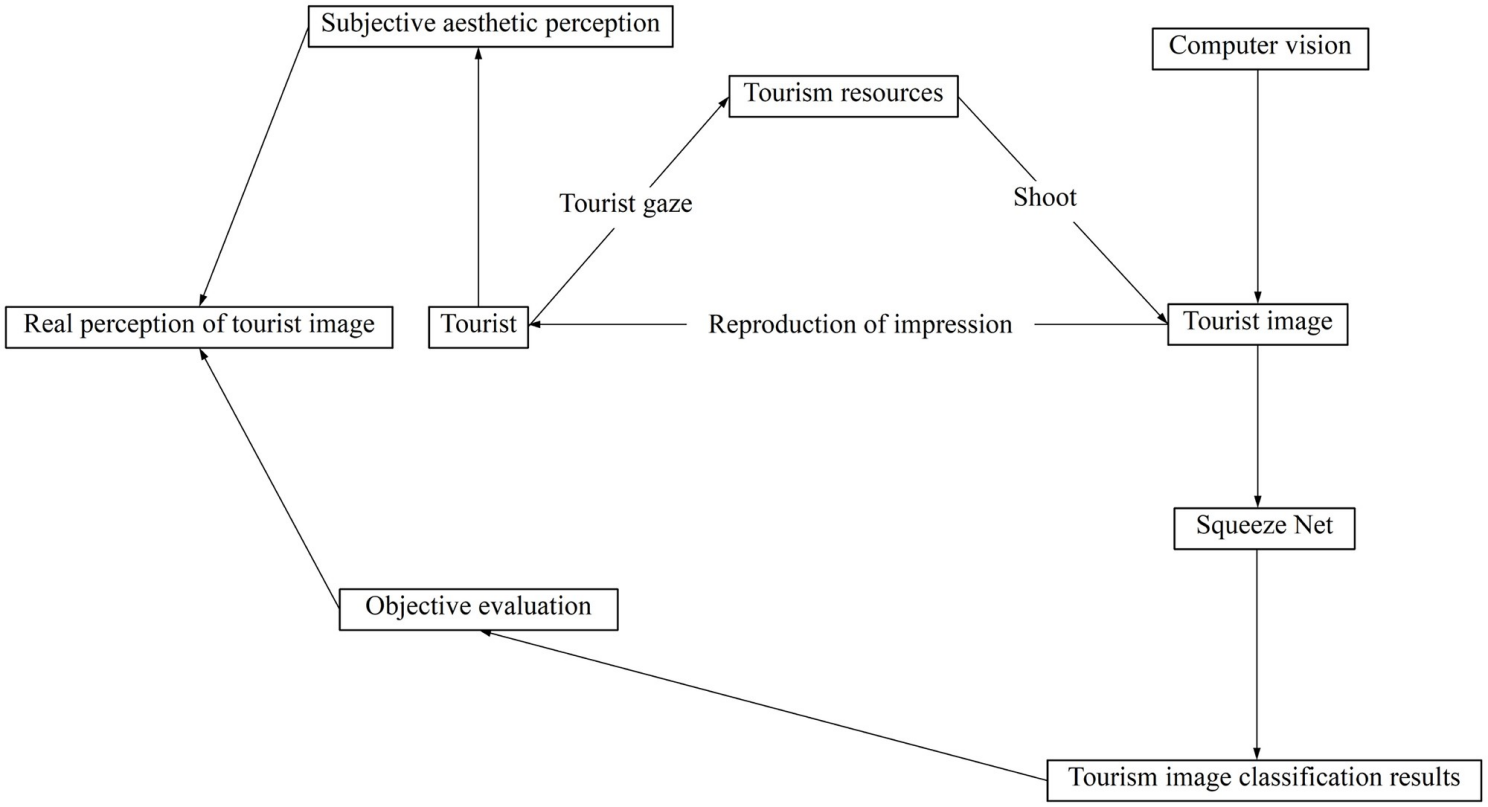
Technical framework.

On the basis of comparing the advantages of several types of CNNs, this study improves SqueezeNet, a lightweight convolutional neural network, to classify the tourism images of Slender West Lake uploaded by travelers on Sina Weibo, Ctrip and other online platforms. Tourists’ aesthetic perception of the tourism resources of Slender West Lake is reflected in the tourism images taken in multiple categories, and the use of SqueezeNet model provides a more accurate and objective evaluation basis for the classification of tourism images of Slender West Lake in the future, which is conducive to clarifying the real perception of tourists on the tourism images, in order to grasp the pulse of tourists. This has reference value for the marketing of local tourism products, and also has theoretical significance and application value for the development and planning of tourism resources, the specific technical framework is shown in Fig 1.

### 2.1 Overview of the research area

Located in the northwest suburb of Yangzhou, Jiangsu Province, Slender West Lake is a renowned garden that was established during the Kangqian period of the Qing Dynasty. It was formerly known as Guarantee Lake and was referred to as Hang West Lake by the poet Wang Hang. With its reputation as the ’best garden in the world’, Slender West Lake covers an area of over 2000 mu, including a water area of more than 700 mu. The tourist area spans 100 hectares and stretches from Shugang Mountain of Daming Temple in the north to Xichun Terrace in the south. The water depth of the lake ranges from approximately 1.5 meters to 2.5 meters. Slender West Lake was awarded the title of a national 5A-class tourist attraction in 2010. It was inscribed on the World Cultural Heritage list in 2014 and identified as a ’Demonstration unit of culture and tourism consumption in Jiangsu Province’ in 2022. The scenic spot often holds lotus Festival, Lamei flower exhibition, chrysanthemum fair and other activities. Visitors can enjoy a variety of delicacies such as crab shell Yellow, Sixi Tangyuan, and scallion shortcake while admiring the beautiful flowers. Furthermore, the ’Two Moon’ night tour offers a great opportunity for tourists to unwind and relax during the summer season. The spring willows and the breeze from Hepu echo each other, creating a dreamy paradise under the moonlit night at the Five Pavilion Bridge. The event will also feature performances such as ’Spring River Flower Moon Night’ and ’18 Send’. The event as a whole showcases the ink painting style of Jiangnan, blending modern technology with classical gardens and integrating the beauty of nature with human activities.

### 2.2 Data source

**Fig 2:**
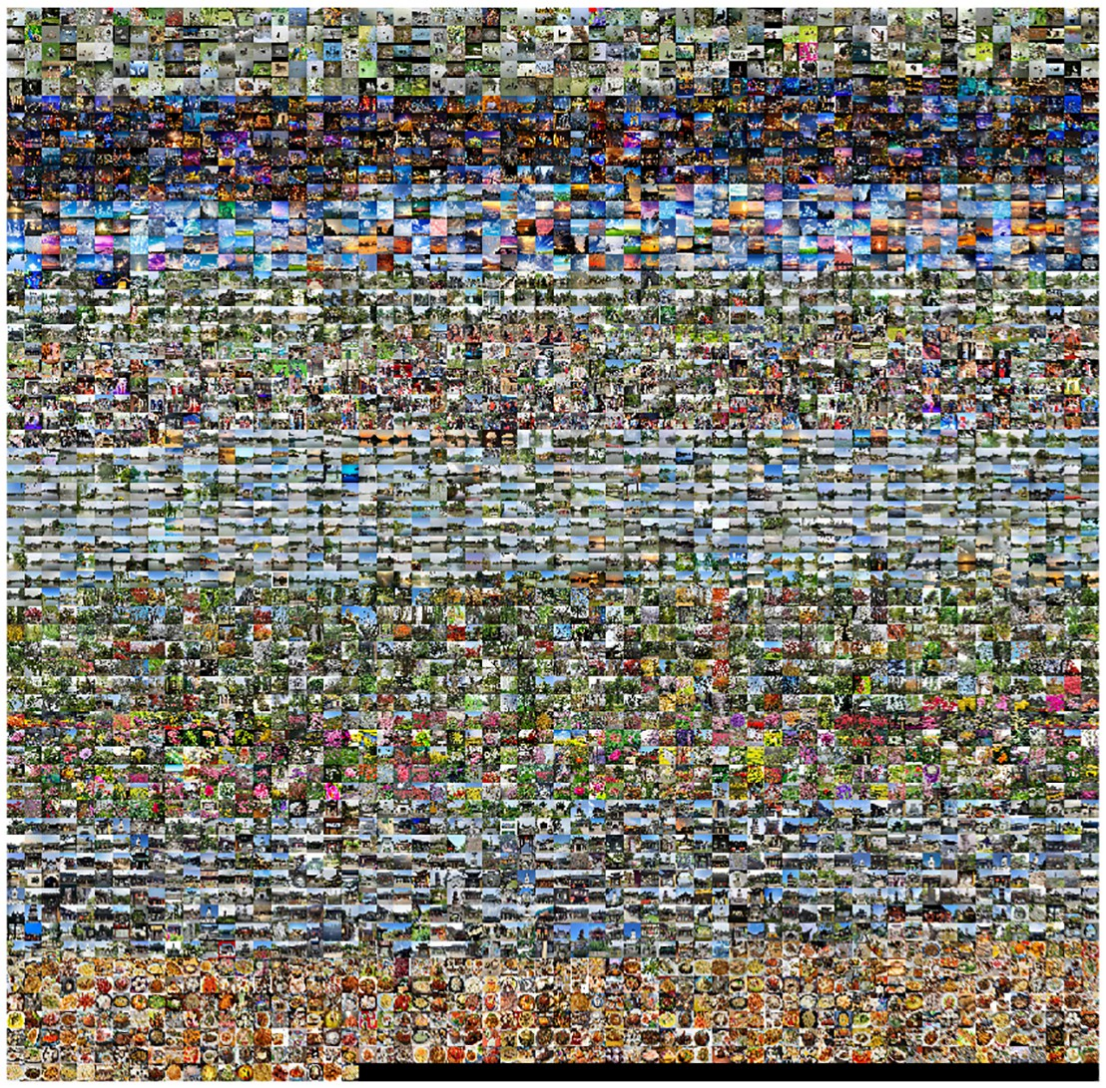
Collection of Slender West Lake tourism image visualization.

This study mainly used Houyi Collector to climb the tourism image data of Slender West Lake from Sina Weibo, Ctrip and other network platforms. The collection rules were set according to the first picture of each user, and a total of 3995 pictures were obtained. The images were then de-weighted using Billfish, which removed 152 duplicates. Then, the images unrelated to the tourist landscape of Slender West Lake, such as screenshot of chat records and emojis, were manually deleted, a total of 103 pictures were deleted. Finally, a total of 3740 tourism images of Slender West Lake were obtained in this study (Fig 2). The image classification is based on the ‘Classification, Survey and Evaluation of Tourism Resources’ (GB/T18972-2017) and the status quo of tourism development of the Slender West Lake. The collected tourism images are divided into ten categories, and the tagged images are put into various folders to form data sets with the help of Billfish software.

**Fig 3:**
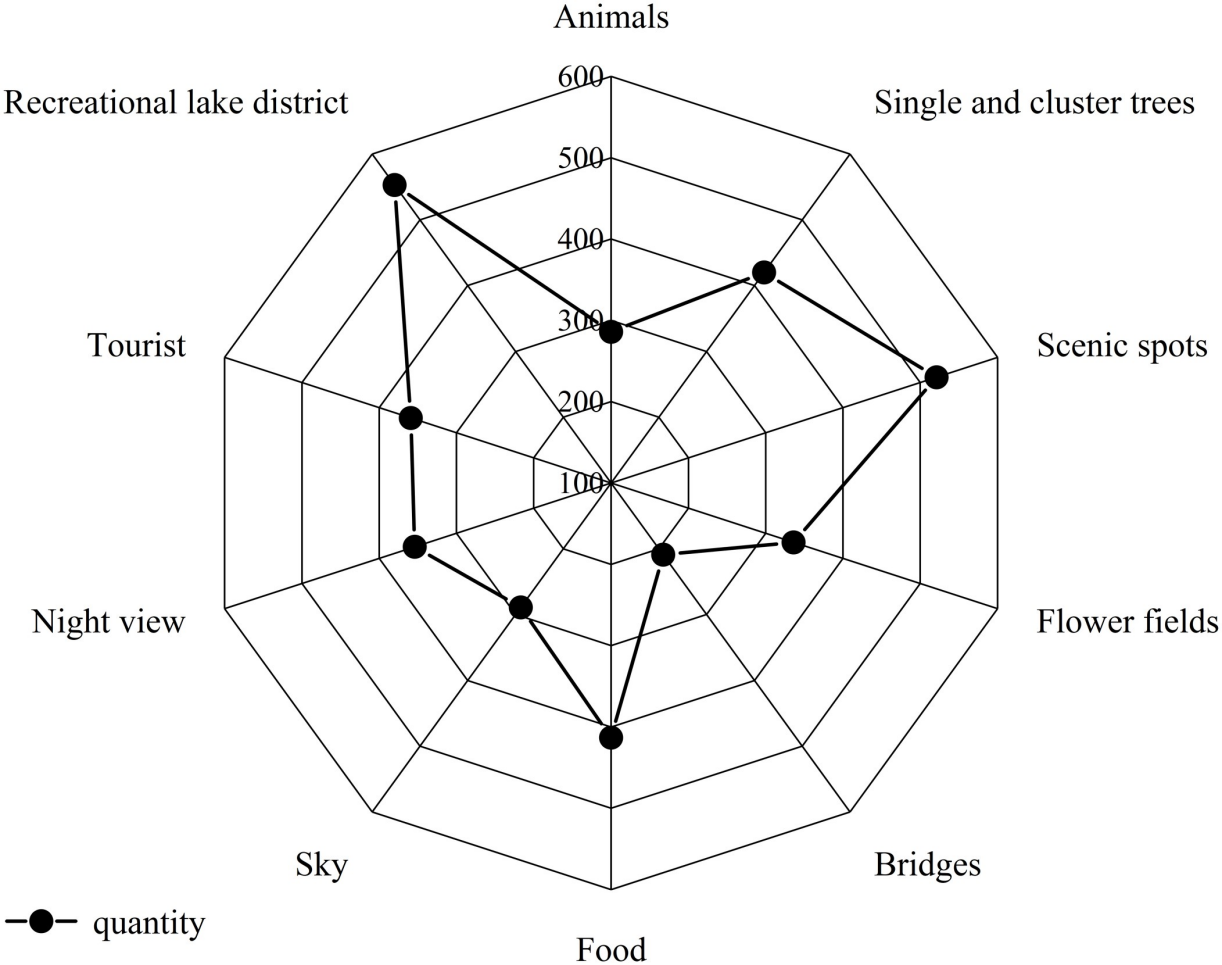
Comparison of the number of ten kinds of images.

These ten categories include 286 images of animals, 420 images of single and clumped trees, 521 images of viewpoints, 336 images of floral lands, 209 images of bridges, 413 images of food, 289 images of the sky, 354 images of night scenes, 359 images of tourists, and 553 images of recreational lakes, with a comparison of the exact number of images shown in Fig 3.

### 2.3 Research method

#### 2.3.1 Gaussian noise

Taking into account the impact of image acquisition equipment, optical quantum density, and other factors on image authenticity, along with the mathematical simplicity of Gaussian noise, this type of noise is commonly referred to as normal noise. For this research, a 0.1 times Gaussian noise was introduced to the preprocessed image. The probability density function of Gaussian noise follows a normal distribution, as illustrated below:

$$p(z)=\frac{1}{\sqrt{2\pi \sigma }}exp\left\{{-(z-\mu )}^{2}/2\sigma^{2}\right\}$$ (I)

In formula, *z* represents the gray value of image element, *μ* is the expectation of *z*, *σ* is the standard deviation of *z*.

#### 2.3.2 Linear spatial filtering

Linear spatial filtering is a commonly used digital image enhancement method. It determines the neighborhood range according to the mask, takes the value in the mask as the coefficient, multiplies the pixel value corresponding to the coefficient in the neighborhood, and then adds it up as the brightness value of the pixel at the central point, which is processed by sliding the center point of the neighborhood through the image. Since digital image is a two-dimensional discrete system, the corresponding convolution operation formula of linear spatial filtering is as follows:

$$y(j,i)=\sum_{m}\sum_{n}h(m,n)x(j+m,i+n)$$ (II)

In formula, (*j*, *i*) represents the spatial position of the image, *y*(*j*, *i*) represents the change position of *x*(*j*, *i*) position of the original image after filtering enhancement processing, *h*(*m*, *n*) represents the unit impulse response of a discrete system.

#### 2.3.3 Convolutional neural network

Convolutional neural networks are generally composed of input layer, convolutional layer, pooling layer, fully connected layer and output layer, in which there is no limit to the number of convolutional layer and pooling layer.

**Fig 4:**
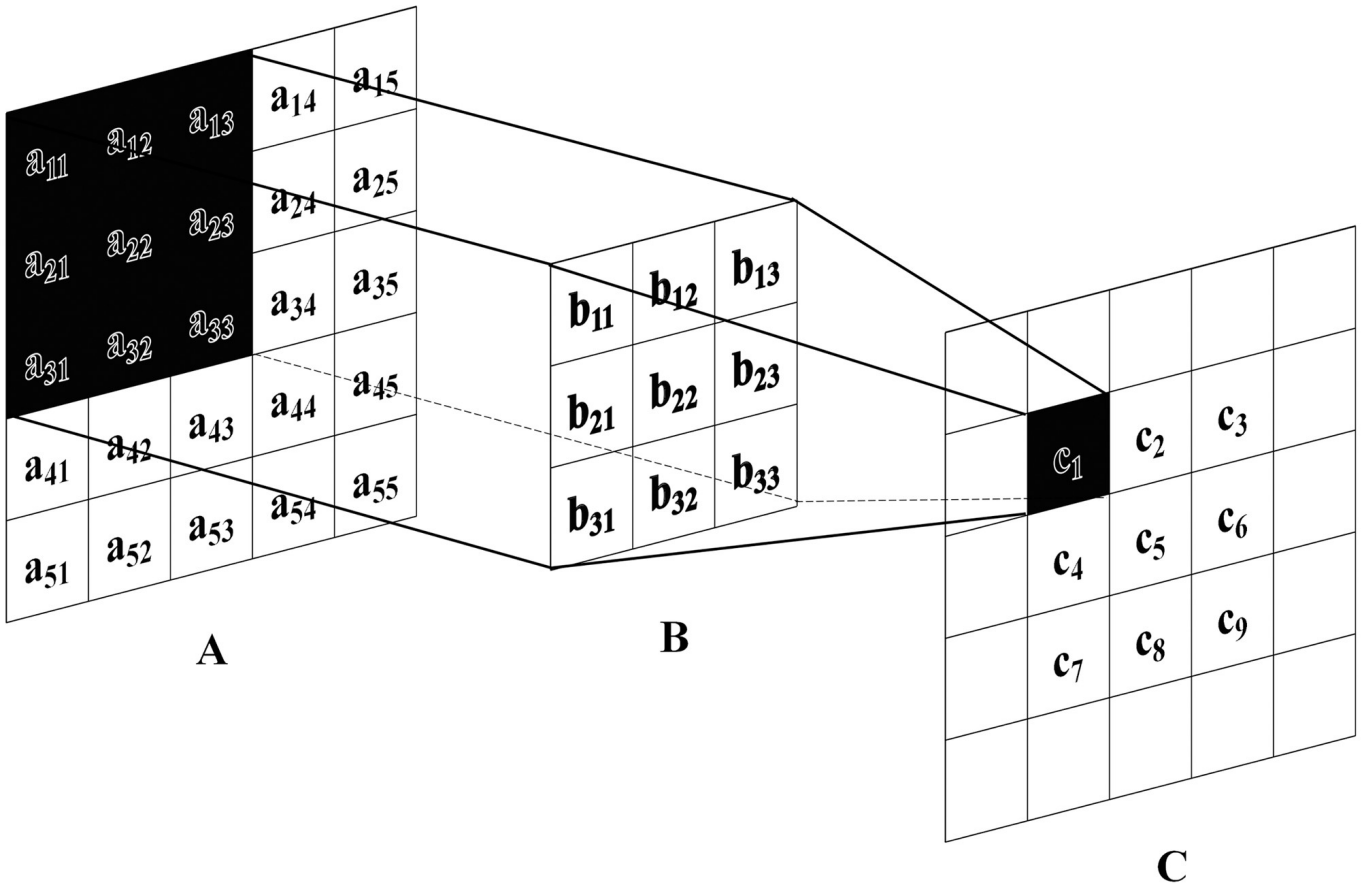
Schematic diagram of convolutional layer operation.

*(1) Convolution layer*. As shown in Fig 4, assuming that the input image is regarded by the computer as the pixel element matrix shown in Figure A, the function of the convolution layer is mainly to extract features from A by using the convolution kernel shown in Figure B, which is specifically manifested in multiplying elements by elements in the convolution kernel B and the corresponding region that it passes through A to output the corresponding pixel element of C in the Figure. This is shown in Fig 4: a_11_b_11_+a_12_b_12_+a_13_b_13_+a_21_b_21_+a_22_b_22_+a_23_b_23_+a_31_b_31_+a_32_b_32_+a_33_b_33_ = c_1_.

*(2) Activation function*. The activation function is used to express the functional relationship between the output of the upper node and the input of the lower node in the multi-layer network. Compared with the traditional saturated nonlinear function, the unsaturated nonlinear function has more advantages when dealing with gradient explosion and other problems. Currently, the unsaturated nonlinear function is commonly used as the activation function in CNN. For example, the ReLU function changes all negative input values to zero and stores positive function values in order to realize unilateral inhibition. The corresponding calculation formula is as follows:

f(x)=max(0,x) (III)

In formula, *x* is the argument to ReLU.

*(3) Pooling layer*. Pooling layer, also known as the undersampling layer, is a process of data compression in convolutional neural networks on the basis of keeping original image features unchanged in order to prevent over-fitting and speed up training. There are two types of pooling layers: average pooling layer and maximum pooling layer. The pooling function uses the overall statistical characteristics of adjacent output at a certain position to replace the network output at that position. The average pooling layer takes the mean value in adjacent rectangular areas, while the maximum pooling layer takes the maximum value.

*(4) Fully connected layer*. Each neuron in the fully connected layer is fully connected with all the neurons in the previous layer, and the local feature information obtained from the convolution layer and pooling layer can be integrated by using the value matrix, and the multi-dimensional vector can be converted into a one-dimensional column vector, so as to realize the classification of data.

*(5) Loss function*. The loss function is an arithmetic function used to measure the degree of difference between the predicted value and the true value of the model, it is a non-negative real-valued function, the smaller the loss function, the better the robustness of the model. The Softmax loss function is more commonly used in CNNs, and its calculation formula is as follows:

$$L_{softmax}=-\frac{1}{N}\sum_{i=1}^{N}log(y_{i})$$ (IV)

Where *y*_*i*_ is the probability value of the image category to which the current sample belongs and *N* is the number of categories of the sample.

## 3 Architecture of convolutional neural network SqueezeNet

The application of convolutional neural networks in image classification can be traced back to LeNet-5 in 1998 [29]. This network was the first to define the basic structure of CNN, which extracted similar features in multiple locations and had the characteristics of automation and few parameters. At that time, it was often used by banks to identify handwritten digits on checks. In 2012, AlexNet, known as the first modern deep convolutional network model, achieved innovation on the basis of predecessors, mainly reflected in the first use of multiple Gpus for training, breaking through hardware limitations. Using ReLU as a nonlinear activation function, the convergence rate of the network model is faster. Parameter optimization strategy is used to enhance CNN learning ability [30]. However, although the VGG convolutional neural network in 2014 reduced the size of the convolutional kernel, it deepened the number of layers of the network and could obtain good image classification results, but it consumed a huge amount of computing resources [31]. GoogLeNet, also proposed by the Google team in 2014, also has a deep network layer, but it only has 5 million users, while AlexNet has 15 times that and VGG three times that. GoogLeNet also exposed some problems, especially in the case of a large number of parameters, if the training data set is limited, it is easy to overfit, which makes the model difficult to apply. However, the problem of large parameters still could not be solved [32]. Thus the creation of the SqueezeNet convolutional neural network in 2016.

**Fig 5:**
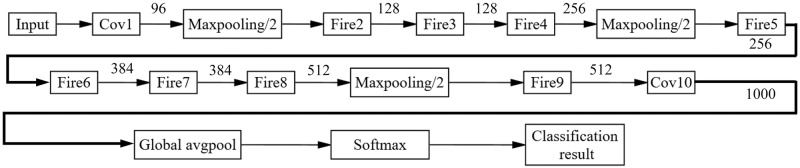
SqueezeNet model structure.

As a lightweight convolutional neural network, SqueezeNet was innovated on the basis of AlexNet. A model with smaller parameters was built on the premise of ensuring the same accuracy. Fire Module was introduced as the basic network unit, and the feature map was compressed and joined in dimension, making the model parameters compressed efficiently. At the same time, the network model is robust. Compared to the original AlexNet, SqueezeNet has 50 times fewer parameters, and the overall model size is less than 0.5 MB. As shown in Fig 5, SqueezeNet starts with a convolutional layer, then adds 8 Fire modules intermingled with 3 maximum pooling layers, Fire9 is followed by a convolutional layer Cov10, and finally an average pooling layer, sorting the output with Softmax functions. This setting greatly reduces the number of parameters and facilitates the mobile terminal to calculate data quickly.

**Fig 6:**
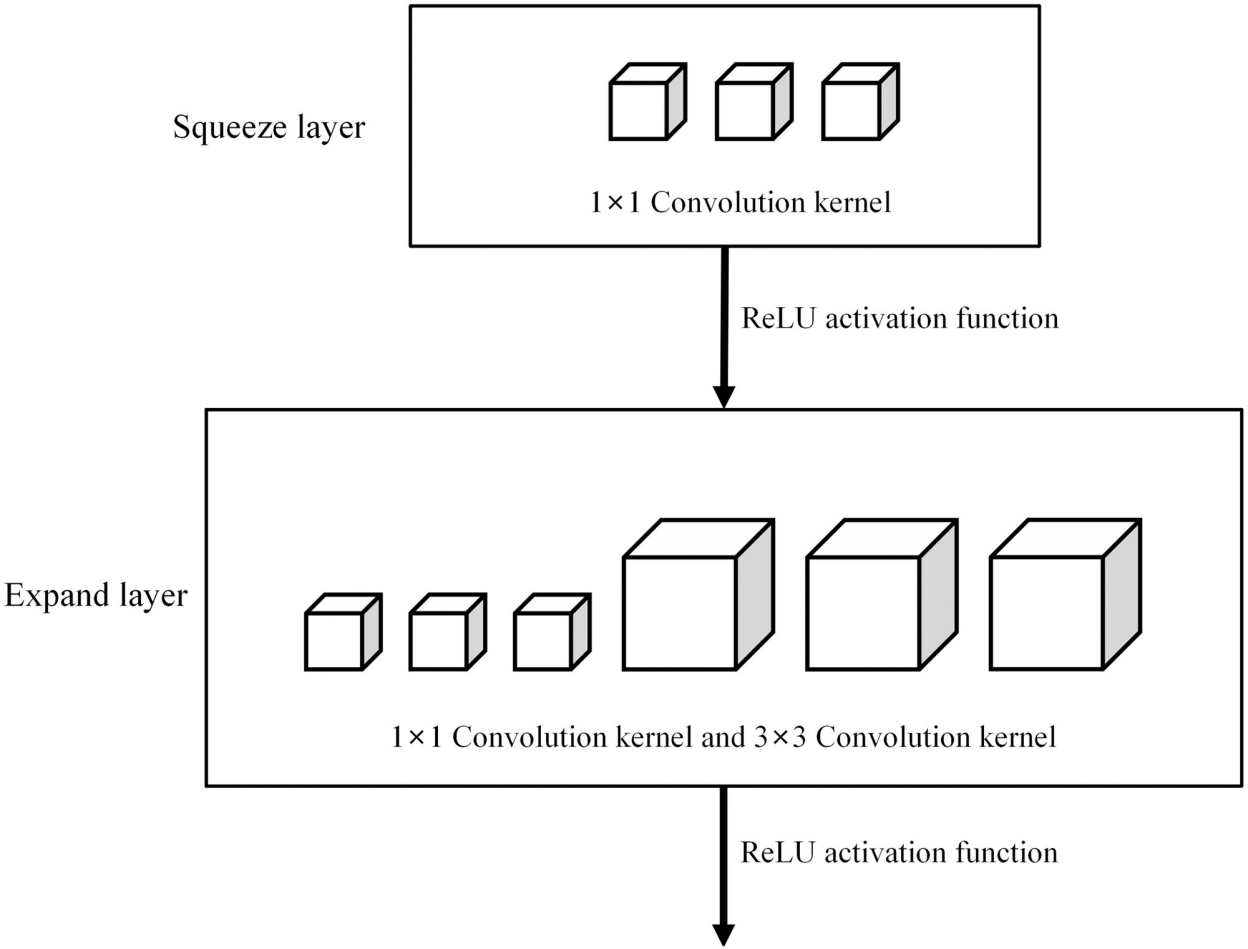
Fire Module structure.

As shown in Fig 6, Squeeze layer and Expand layer exist in the Fire Module of SqueezeNet, and both layers use ReLU activation function when output. Squeeze contains 1×1 convolution nuclei, the number of which is m_1_n_1_. The Expand layer contains two sizes of 1×1 and 3×3 convolution kernels. The number of 1×1 convolution kernels is represented by q_1_n_1_. And the number of 3 by 3 convolution kernels is q_3_n_3_. In this structure, m_1_n_1_<q_1_n_1_+q_3_n_3._

In related studies, some scholars have applied SqueezeNet in practice and even compared it with other convolutional neural networks. Chunlong Wang et al. graded peanut pod quality based on the improved SqueezeNet and achieved 97.83% accuracy [33]. Wang Yiding et al. proposed an improved double-channel algorithm based on SqueezeNet to study the microscopic feature map of 15 Chinese medicinal materials powder, with an accuracy of 90.33% [34]. Cheng Guojian et al. used SqueezeNet for rock image classification, and its verification set accuracy reached 90.88%, which was significantly higher than AlexNet and GoogLeNet et al. and the size of SqueezeNet model was only 4.78 MB [35]. Therefore, SqueezeNet plays a continuing role in the study of image classification, and its classification effect and model size are generally superior to those of other CNN.

## 4 Experiment and result analysis

### 4.1 Experimental environment

The experiment was conducted on a computer with Intel(R)Core(TM)i5-3230M processor, NVidia GeForce GT 720M graphics card, and 4GB of memory. The computer operating system is Windows10 version 20H2. The model running platform is Matlab 2021b.

### 4.2 Image preprocessing

**Fig 7:**
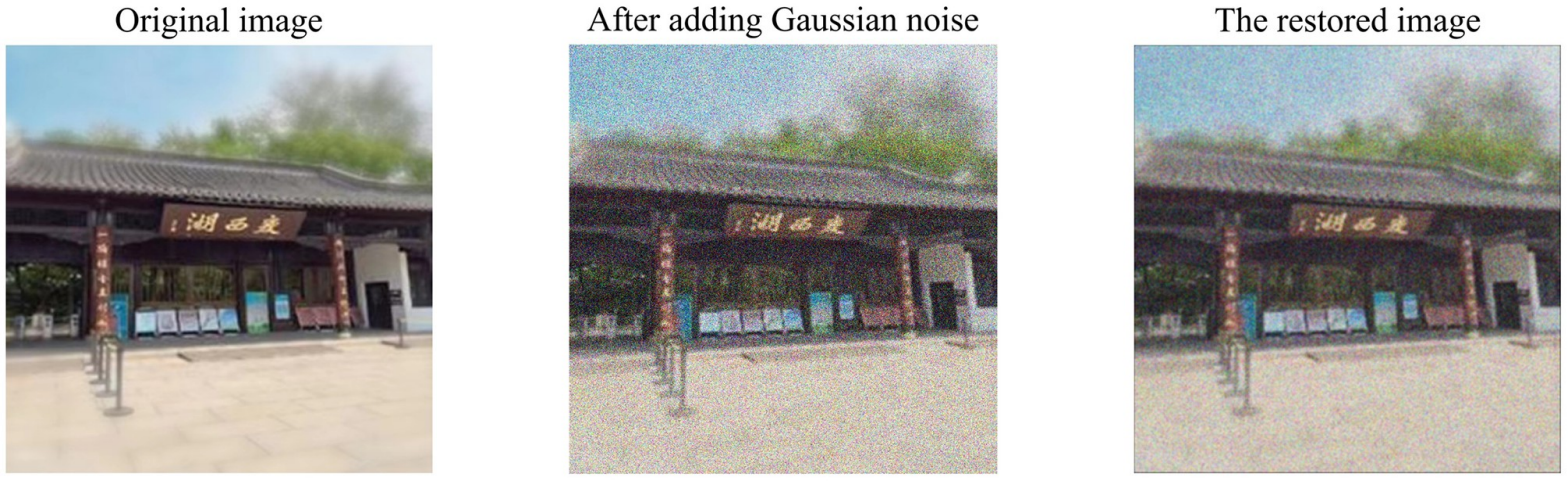
Example of fuzzy image preprocessing.

In this study, the resolution of 3740 images of Slender West Lake tourism is generally high. However, objective factors such as optical quantum density, electrical and mechanical motion, and the circuit of the internal equipment of the system will interfere with the image to form noise and cause image distortion during image formation, transmission or transformation [36], so there are still some fuzzy images in the data set. Considering the complex content of tourism images and the difficulty of computer recognition caused by fuzzy images, this study used Matlab 2021b to preprocess fuzzy images before the experiment. In the process of processing, 0.1 times Gaussian noise is added to each image to make the original noise of the fuzzy image obey Gaussian distribution. After that, linear spatial filtering is adopted to de-noise the digital image to achieve the effect of digital image enhancement, which is convenient for the computer to distinguish the category of tourism image more quickly in the subsequent experiment, as shown in Fig 7.

### 4.3 Experimental process

**(1) Model construction.** The model of this study was derived from the existing SqueezeNet network in Matlab2021b deep network designer, and improved on this basis. A two-dimensional convolution layer named Covnn was added after Fire9 module SqueezeNet, and the size of its filter convolution kernel was set to 1×1. The number of convolution cores is set to 10, and then the original two-dimensional convolution layer Cov10 of the model is renamed to Covnew, and the setting similar to that of Covnn is carried out. The difference is that the weight learning rate and deviation learning rate in Covnew are set to 10, which makes the learning speed of the new layer faster than that of the transfer layer. Finally, the original output size of 1000 classification layer is replaced by the automatic output of the new classification layer. The new SqueezeNet network has been analyzed by Deep Learning Designer with no warnings and no errors.

**(2) Image data import.** In this study, 3740 tourist image data sets of Slender West Lake were imported, 70% of the images were used as training data set and 30% were used as verification data set, and all images were unified into 227×227 sizes to fit the setting of SqueezeNet model. At the same time, random rotation from [–90,90] degrees on the X-axis and random rescale from [1,2] can help to memorize the precise details of the training image and prevent overfitting of the network.

**(3) Algorithm description.** In this experiment, an improved SqueezeNet model was used for training. The stochastic gradient descent optimization algorithm and the unsaturated nonlinear activation function ReLU were used to train the model. dropout technology is also used to prevent overfitting, and the parameter is set to 0.5, meaning 50% of the neurons are deleted. The initial learning rate was set as 0.0001 and momentum as 0.9 to describe the decline trend of loss function value in training. In this study, the maximum Epoch was set as 8, that is, the training rounds were set as 8 rounds. The Min Batch Size is set to 20, that is, 20 samples for each training. The Validation Frequency between the validation measures is set to 60. After undergoing computer training, image data is first extracted by the convolution layer, then undersampled by the maximum pooling layer, and then received by the Fire Module for further dimensionality reduction, which greatly reduces the number of parameters. After further feature extraction by the last two convolution layers, all spatial information is integrated by the average pooling layer. The output is matched with the Softmax classifier.

**Fig 8:**
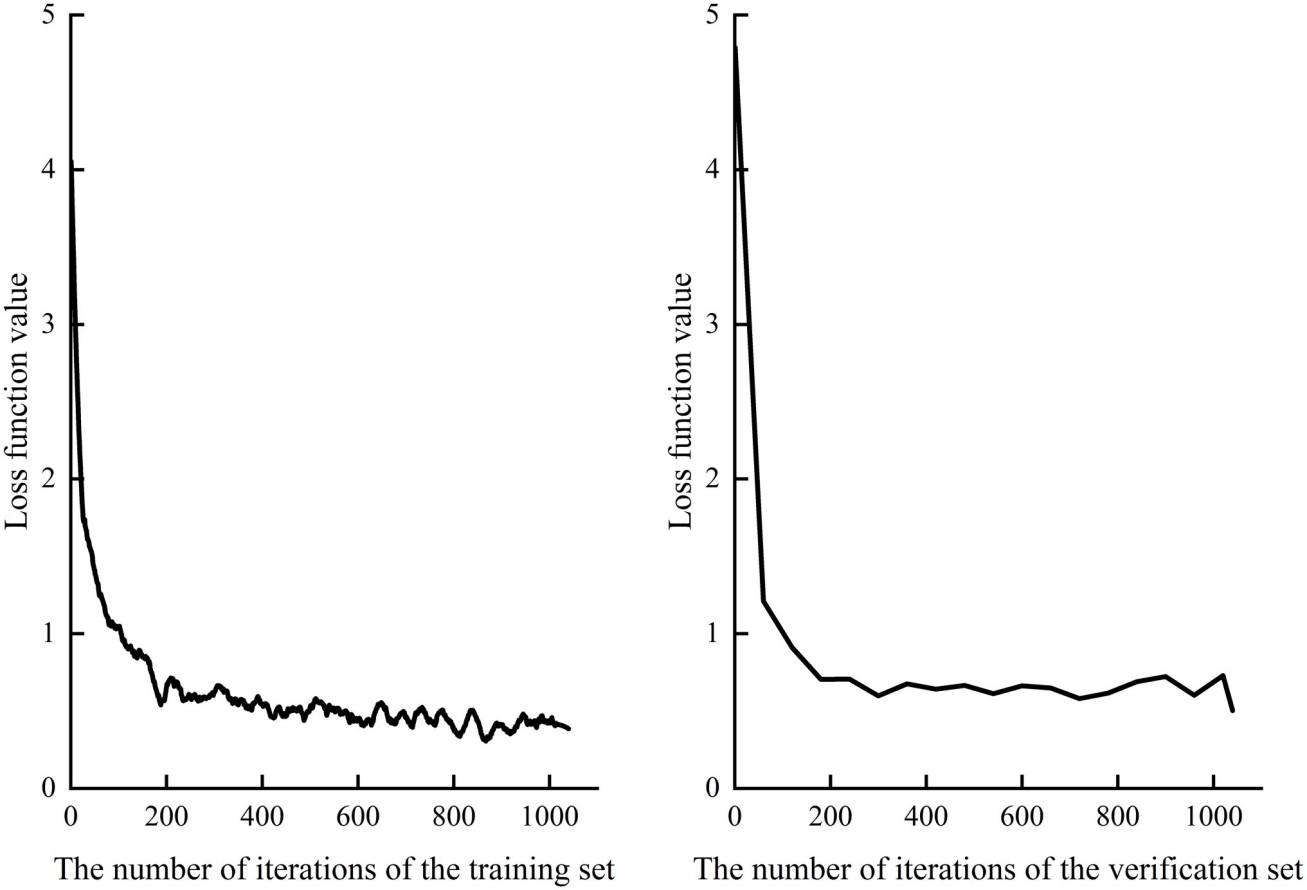
Loss function on training set and validation set.

**Fig 9:**
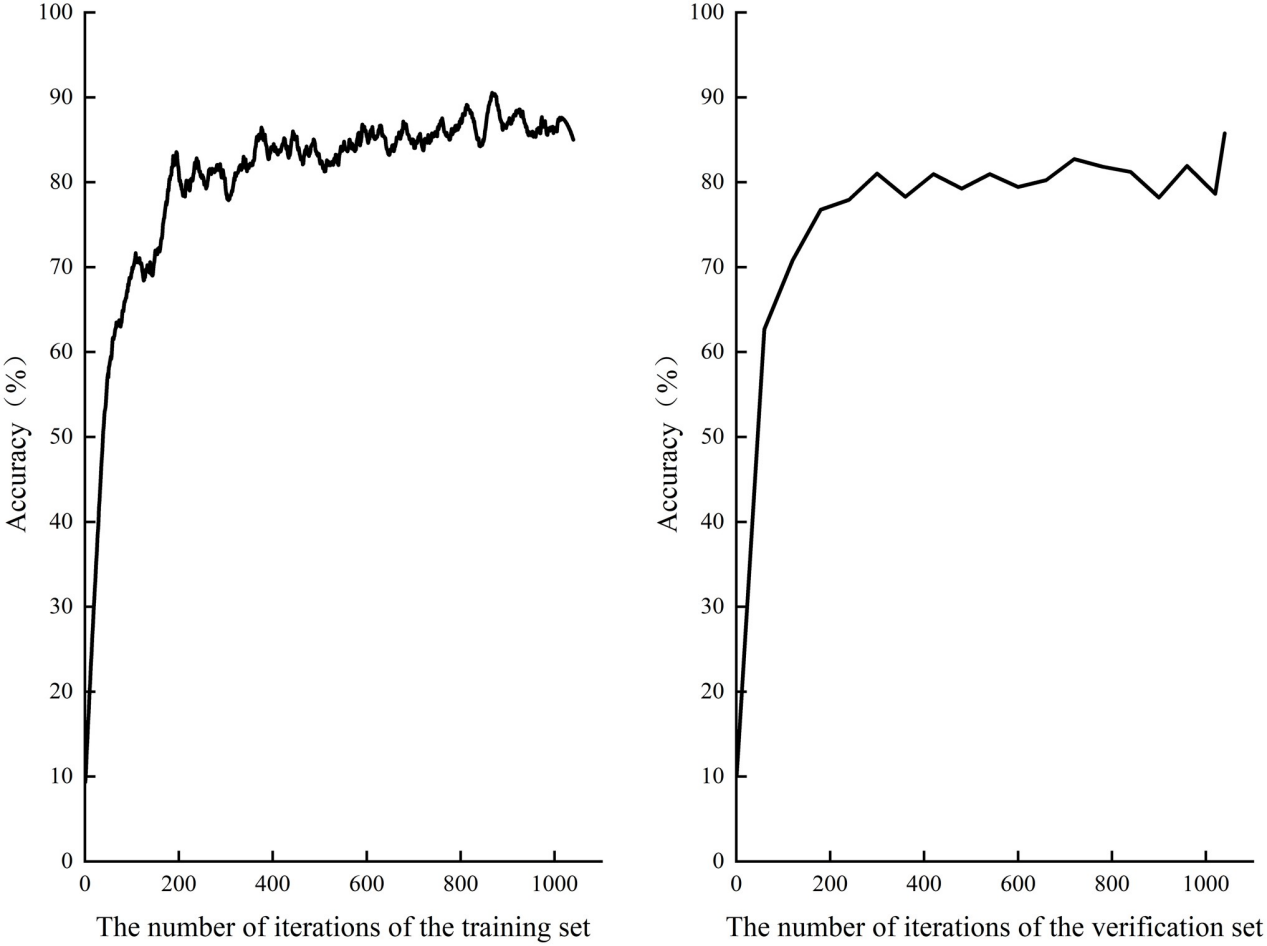
Accuracy on training set and verification set.

**(4) Experimental result.** As the experimental environment is a single CPU with limited configuration, the initial learning rate is set small, and the complexity of tourism images and other factors increase the calculation difficulty, the experiment has experienced 1040 iterations. Fig 8 shows the loss function curve of training set and verification set, and Fig 9 shows the accuracy curve. As can be seen from the figure, although the loss function curve and the accuracy curve both fluctuate to varying degrees, the overall trend of the two types of curves is good. Among them, the classification accuracy of the training set is up to 90%, the classification accuracy of the verification set is up to 85.75%, and the model size is only 2.64 MB. This proves that the improved SqueezeNet model in this study ensured the high-precision classification of tourist images of Slender West Lake with a few parameters. More importantly, the training model can not only be further applied in the future Slender West Lake tourism image classification, but also can be deployed and used on embedded devices.

**Fig 10:**
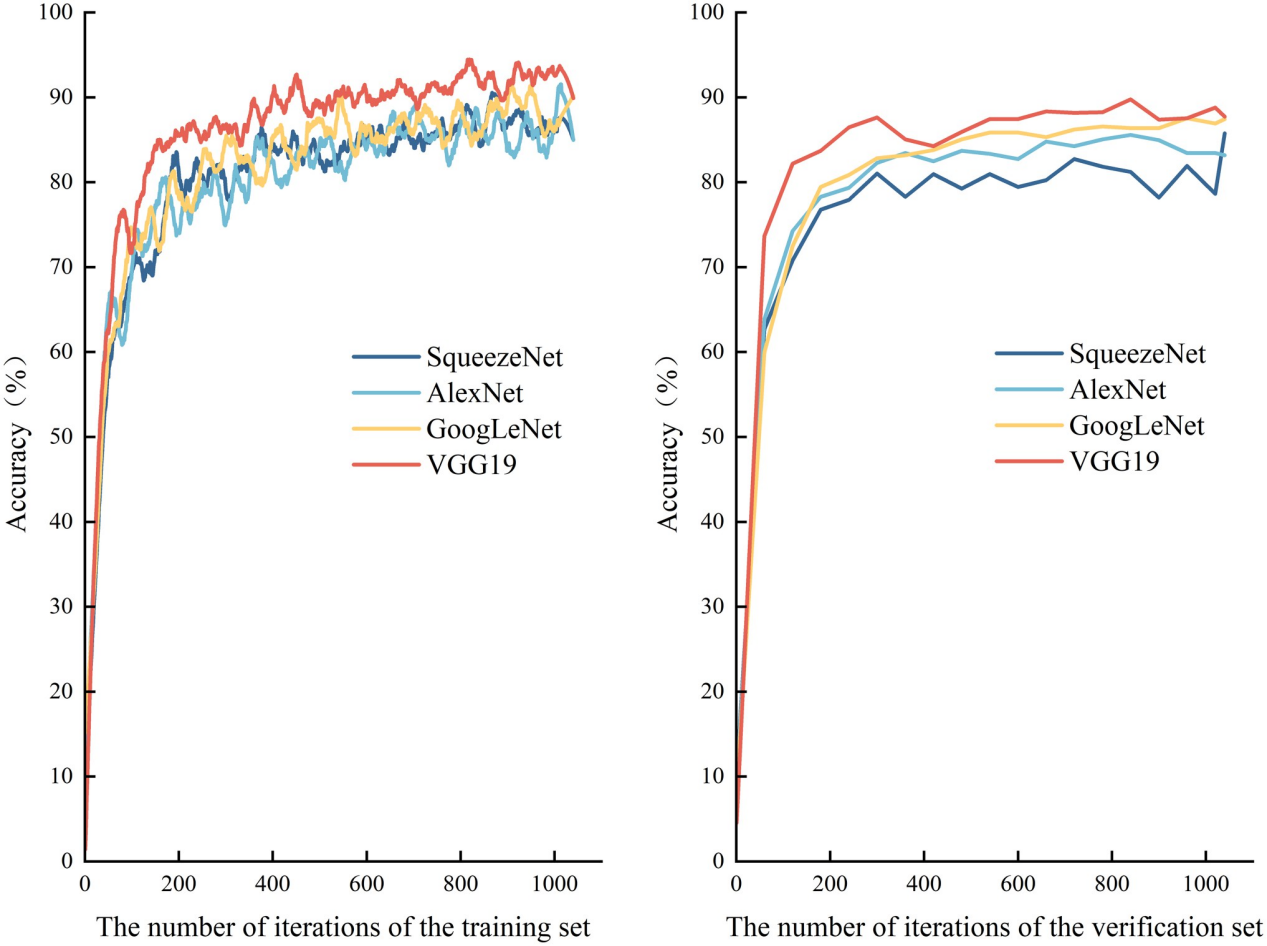
Accuracy on training and validation sets.

**(5) Model comparison results.** This comparison experiment compares the SqueezeNet model with AlexNet, GoogLeNet, and VGG19, and the accuracies of the specific network models on the training and validation sets are shown in Fig 10. With the same number of iterations, the classification performance of these classes of models is readily apparent. After about the 200th iteration, the accuracies of the above classes of models are maintained at a high level and show varying degrees of fluctuation in the subsequent hundreds of iterations time period, but generally stabilize. The classification effect of the VGG19 model on the validation set is more significant, with an accuracy of 87.71%, while the classification accuracy of GoogLeNet is also as high as 87.44%. In contrast, the SqueezeNet model’s accuracy is also not weak, up to 85.75%, which is better than the AlexNet model’s accuracy of 83.17%.

In addition to the trend of the accuracy of each type of model shown in Fig 10, more importantly, as shown in Table 1, the overall size of the SqueezeNet network model after training is only 2.64 MB, and such a parameter is much smaller than that of other models. As far as the training time is concerned, the SqueezeNet model took only 74 minutes and 27 seconds to be trained in this study of Slender West Lake tourism image classification, which is significantly faster than other models. Therefore, compared with other models, the SqueezeNet convolutional neural network model in this study can reduce the number of parameters to a greater extent, while ensuring high accuracy image classification performance, and at the same time conduct faster image classification training, which can be called a high-quality and high-efficiency network model. The advantages of the SqueezeNet network model can be used in the application of the mobile devices to bring more significant benefits to the tourism researchers. tourism researchers more significant inspiration and also provide help in analyzing tourist destination image perceptions and thus promoting and developing tourism destination resources.

**Table 1:** Comparison table of various types of deep network models.

Model	Accuracy /%	Model size /MB	Training duration
**SqueezeNet**	**85.75**	**2.64**	**74 minutes, 27 seconds**
AlexNet	83.17	216	81 minutes, 37 seconds
GoogLeNet	87.44	24.9	239 minutes, 53seconds
VGG19	87.71	510	738 minutes, 23 seconds

## 5 Conclusion

In this paper, a classification study was carried out on 3740 collected Slender West Lake tourism images based on SqueezeNet convolutional neural network model, and the specific conclusions are as follows.

1. In this study, the SqueezeNet convolutional neural network model is improved to classify complex tourism images under a limited experimental environment, and its validation accuracy is as high as 85.75%, and the model size is only 2.64 MB. Because tourism images involve tourists, tourism resources, and other complex elements, there are greater difficulties for computers to perform the operations of convolution kernel extraction of feature maps and other operations. Because tourism images involve tourists, tourism resources and other complex elements, it is difficult for computers to perform operations such as extracting feature maps with convolutional kernels. The experimentally trained model combines the advantages of high accuracy and few parameters, which can not only be used for the classification of tourism images of Slender West Lake, but also be used for updating mobile clients or deploying embedded devices, which is convenient for scholars engaged in the research related to tourism images.
2. Most of the previous researches related to tourists’ experience perception use tourists’ online comments as references, or use questionnaires and interviews to obtain research data in order to approximate the real perception of tourists. Tourism image is a reproduction of the emotional impression of tourists when they gaze at tourism resources, and it is one of the bases for tourism marketers and tourism planners to judge the attractiveness of tourism resources to tourists. However, tourism image research by human vision alone is subjective and difficult to quantify, and batch processing of images is also a difficult task for the human eye to accomplish. Therefore, in this study, computer vision is utilized instead of human vision to classify tourism images, which enables subsequent studies to further explore the real perception of tourists with reference.
3. The multi-dimensional aesthetic perception of tourists cannot be fully simulated by computers, while convolutional neural network as a simulation tool can provide objective evaluation for image classification in this study, thus maximizing the reduction of distortion caused by the subjective aesthetic perception bias of tourists, but it cannot completely replace the tourists’ complex aesthetic evaluation of tourism resources. Tourism image research is not only limited to this aspect of image classification, tourism image segmentation and other research also has a plastic space, but whether it is image classification or image segmentation are only research tools, are serving the scientific means of tourism research. Therefore, the way of computer vision instead of human vision in this study is only a way of tourism image research, and the root of the problem lies in the limitations of human vision, and computer vision just makes up for this deficiency. Therefore, in the future tourism image research should be based on tourist vision and supplemented by computer vision. In the future, research other than tourism image can also be combined with artificial intelligence, and the future of tourism can be vigorously developed with the aid of new technology.

## Supporting information

S1 File (XLS)
